# Sedation With Dexmedetomidine in Critically Ill Burn Patients Reduced Delirium During Weaning From Mechanical Ventilation

**DOI:** 10.7759/cureus.31813

**Published:** 2022-11-23

**Authors:** Bianca Stangaciu, Stavros Tsotsolis, Sophia Papadopoulou, Athina Lavrentieva

**Affiliations:** 1 Intensive Care Unit, Achilopoulio General Hospital of Volos, Volos, GRC; 2 Faculty of Health Sciences, School of Medicine, Aristotle University of Thessaloniki, Thessaloniki, GRC; 3 Plastic Surgery, General Hospital of Thessaloniki "George Papanikolaou", Thessaloniki, GRC; 4 Intensive Care Unit, General Hospital of Thessaloniki "George Papanikolaou", Thessaloniki, GRC

**Keywords:** sedation, mechanical ventilation, delirium, burns, dexmeditomidine

## Abstract

Introduction: Weaning of mechanical ventilation while maintaining appropriate pain control and preventing delirium is one of the most challenging aspects of burn care. Dexmedetomidine, an α_2_-adrenergic receptor agonist used for sedation may improve intensive care unit (ICU) patients’ arousal status and enhance patient comfort.

Objectives: To determine the efficacy of dexmedetomidine vs. standardized usual care (midazolam or propofol) in maintaining sedation and reducing delirium in burn patients while weaning off mechanical ventilation.

Material and methods: A total of 56 mechanically ventilated patients who fulfilled the criteria for weaning were enrolled in the study. Group 1 (26 patients) received dexmedetomidine 1 mcg/kg over 15 minutes as a loading dose, followed by 0.4-0.1 mcg/kg/h. Group 2 (30 patients) received usual sedation with midazolam 0.08 mg/kg/h or propofol 15- 30 mcg /kg/min).

Results: Dexmedetomidine was not associated with a significantly shorter duration of mechanical ventilation (Mean {IQR}: 9.3 {4,12} versus 7.5 {4,10}, p=0.3). Patients who received dexmedetomidine had a lower delirium rate (38,4% on Day 1 to 7,7% on Day 5) in comparison with patients from the usual care group (53,3% on Day 1 to 20% on Day 5) during the five days after the onset of weaning process (p=0.02) and had less need for supplemental use of analgesia (23.1% versus 53.3%, p=0.045) and antipsychotic agents (15.4% versus 53.3%, p=0.01). The most notable adverse effect of dexmedetomidine was bradycardia.

Conclusions: Dexmedetomidine may provide effective light sedation and is associated with fewer sedation-related adverse effects in burn patients. Sedation with dexmedetomidine during the weaning process in adult burn patients was associated with lower delirium rates, a trend towards the earlier withdrawal of mechanical ventilation but did not seem to improve the total duration of mechanical ventilation.

## Introduction

Sedation and analgesia are considered vital components of intensive care practice in critically ill burn patients who are receiving mechanical ventilation, however, the choice of sedative and analgesic agents is of great importance and can affect the outcome of these patients [1].

Different factors influence the choice of sedative drugs including the severity of patients’ conditions, the need for prolonged mechanical ventilation, availability, and the overall costs of a particular sedative agent. The recent recommendations of the Society of Critical Care Medicine regarding pain agitation/sedation, delirium, immobility, and sleep disruption highlight the need for assessment of the sedation level, the need for establishing specific sedation goals for each patient, and suggest using the lightest possible sedation avoiding benzodiazepines and using non-benzodiazepine sedatives (either propofol or dexmedetomidine) as first-line drugs [2].

Dexmedetomidine, a high-affinity selective α2-adrenoceptor agonist has advantages over conventional sedatives, since it has a good analgesic effect while causing less respiratory depression, allowing patients to be more interactive and therefore promoting early weaning from mechanical ventilation [3].

Studies published in the last decade demonstrated that the use of dexmedetomidine could be associated with a reduced length of intensive care unit (ICU) and hospital stay, reduced duration of mechanical ventilation and shorter time to extubation, increased number of days free from coma, reduction of the supplemental dose of sedative or analgesic drugs and a lower mortality rate in critically ill patients [4,5]. Dexmedetomidine is considered a reliable treatment option for the prevention and treatment of delirium in ICU patients which could facilitate faster weaning from mechanical ventilation when compared with conventional sedation agents [6,7]. A number of publications support the advantageous profile for sedation and analgesia in intubated and mechanically ventilated burn patients during treatment in the intensive care unit and sedation prior to and/or during burns debridement and dressings or other procedures on non-intubated burn patients [8-14]. Dexmedetomidine has an acceptable tolerability profile; with hypotension, hypertension, and bradycardia being the most commonly reported adverse reactions [15].

Our study aims to evaluate the effectiveness and safety of dexmedetomidine for sedation of Burn ICU patients and to investigate whether this agent improves outcomes when compared to other commonly used sedative drugs (propofol and midazolam). Clinical data of patients who received sedation with the use of either dexmedetomidine or midazolam and propofol during the weaning phase from mechanical ventilation were evaluated. We tested the hypothesis that dexmedetomidine would facilitate the early extubation of burn patients from mechanical ventilation, reduce the risk of delirium, and reduce the need for supplemental analgesia.

## Materials and methods

Our study was a unicentric clinical trial conducted among Burn ICU patients. The trial was conducted in the Burn ICU of the General Hospital of Thessaloniki "George Papanikolaou" between March 2016 and December 2020. The study was approved by the Institutional scientific review board of the General Hospital of Thessaloniki "George Papanikolaou" (Approval No: 1361/20.11.2019).

All selected patients receiving invasive mechanical ventilation who were successfully weaned from mechanical ventilation were retrospectively included in this study. Patients were divided into 2 groups: Group 1, receiving dexmedetomidine during the weaning process and Group 2 receiving usual care with midazolam or propofol while weaning from mechanical ventilation. Demographic and clinical data of patients allocated to these groups were compared. Collected demographic data included age, total body surface area % that is affected by a burn (TBSA %), abbreviated burn severity index ABSI, and the length of mechanical ventilation. The primary endpoint was the duration of the weaning process (time from the decision to wean from mechanical ventilation to successful withdrawal of mechanical ventilation). Secondary endpoints include: the percentage of patients with a satisfactory sedation score (defined as a Richmond Agitation-Sedation Scale {RASS} score of −2 to +1), the presence of delirium assessed by the use of the Confusion Assessment Method for the ICU (CAM-ICU) method, the requirement for additional analgesia (use of fentanyl), the requirement for the use of antipsychotic medications (haloperidol, risperidone, quetiapine), presence of adverse effects, presence of organ dysfunction assessed by daily Sequential Organ Failure Assessment (SOFA) score, and lengths of ICU and hospital stay.

The sedation level was estimated by using Richmond Agitation Sedation Scale (RASS) and the Delirium assessment was performed according to the Confusion Assessment Method for the ICU (CAM-ICU) [16,17]. The severity and extent of burn injury were estimated and expressed as the total percentage of body surface area (TBSA) that is affected by a burn and the Abbreviated Burn Severity Index (ABSI) [18].

Patients were excluded if they had the following conditions: (1) severe liver disease (Child-Pugh class B or C); (2) acute myocardial infarction or severe heart failure (New York Heart Association functional class 4); (3) second- or third-degree heart block, (4) alcoholism or drug dependence, (5) known mental illness or severe cognitive dysfunction, (6) known allergy to dexmedetomidine.

The χ2 test was used for the comparison of categorical variables and the Wilcoxon rank sum test for the comparison of continuous variables. Statistical tests were 2-sided, and p<0.05 was considered as a benchmark for statistical significance.

## Results

The data of 61 patients with burn injuries who fulfilled the criteria for weaning from mechanical ventilation were analyzed. Seven patients were excluded from the study: two patients suffered from severe heart failure, three patients had drug and alcohol dependence, one patient had chronic liver failure and one patient had severe cognitive dysfunction. A total of fifty-six mechanically ventilated patients who fulfilled the criteria for weaning were enrolled in the study. Group 1 received dexmedetomidine 1 mcg/kg over 15 minutes as a loading dose, followed by 0.4-0.1 mcg/kg/h. Group 2 received midazolam 0.08 mg/kg/h and propofol 15-30 mcg /kg/min), with doses adjusted by bedside nurses to achieve target sedation goals set by clinicians according to the Richmond Agitation-Sedation Scale (RASS, on which scores range from −2 to 1). The χ2 test was used for the comparison of categorical variables and the Wilcoxon rank sum test for continuous variables.

Patients in the dexmedetomidine group were started on a dose of 0.5 μg/kg/h. The mean maintenance infusion dose for dexmedetomidine in Group 1 patients was 0.72±0.4 mcg/kg/h, (mean ± SD); Group 2 patients received routine sedation with midazolam 0.086±0.025 mg/kg/h, (mean ± SD) or propofol 22±5 mcg /kg/min (mean ± SD). According to our unit’s protocol, as mentioned above, doses were adjusted by bedside nurses to achieve target sedation goals set by clinicians corresponding to the target Richmond Agitation-Sedation Scale (RASS), levels ranged from -2 to 1.

No patient deceased during the weaning process and all were successfully weaned from mechanical ventilation. The median duration of infusion of dexmedetomidine was 8 days (2.0 to 8.0, IQR, interquartile range); the medial duration of midazolam and propofol infusion was three days (2-4, IQR) after the decision to wean from mechanical ventilation has been made. The median RASS score in all patients was −1.0 (-2.5 - 1.8, IQR,). Patients from the dexmedetomidine group (Group 1) continued drug administration for a mean of three days (interquartile range, 1.5- 5) after withdrawal of mechanical ventilation. The median SOFA score prior to deciding to begin the weaning process from mechanical ventilation was low, possibly reflecting a hypothetically satisfying likelihood for successful weaning; however, a high percentage of patients from both groups presented agitative delirium at the onset of the weaning process. The baseline characteristics of study participants are depicted in Table 1. Patients in both groups had similar demographic and clinical data; however, dexmedetomidine patients were younger and had a higher percentage of 3rd-degree burn injuries. 

**Table 1:** Participants’ demographic and clinical characteristics Abbreviations: APACHE II, Acute Physiology and Chronic Health Evaluation II; SOFA, Sequential Organ Failure Assessment; TBSA, total percentage of body surface area; the ABSI, Abbreviated Burn Severity Index; IQR, interquartile range.

Variable	Group 1 (n=26)	Group 2 (n=30)	p-value
Age, mean (SD), years	40.4 ±15.3	55.13 ±20.5	0.062
Male, No. (%)	20 (76.9)	22 (73.3)	0.2
ABSI	7.15±0.678	6.4 ±0.496	0.37
TBSA	34,31 (±4,8)	25,00 (±5,7)	0.058
3^rd^ degree burn	12,00 (±3,9)	7,73 (±3,3)	0.026
APACHE II score on admission (median, IQR)	6.8 ±0.6	8.0±0 .9	0.3
SOFA scores on admission (median, IQR)	3.5±1.3	4±1.8	0.3
Charlson comorbidity index on admission (median, IQR)	1 (1.1)	1.2(1)	0.34
Agitated delirium diagnosis, prior weaning (%)	53.8	66.7	0.2
Duration of sedation prior to weaning, days (median, IQR)	7,1(3-9)	6.8 (3-8)	0.2

Patients in the dexmedetomidine group demonstrated marginally higher RASS scores. The use of dexmedetomidine was associated with a slightly lower median duration of the weaning process (from the decision to wean to the withdrawal of mechanical ventilation). However, dexmedetomidine was not associated with a significantly shorter overall duration of mechanical ventilation. Patients in the dexmedetomidine group demonstrated lower requirements for supplemental analgesia and antipsychotic agents. Finally, it is important to acknowledge that although not statistically significant, the duration of ICU stay was longer amongst patients receiving dexmedetomidine. Clinical characteristics of patients during the weaning process are presented in Table 2.

**Table 2:** Clinical data of patients during the weaning process Table 2. Clinical data of patients during the weaning process. Abbreviations: SOFA, Sequential Organ Failure Assessment; RASS, Richmond Agitation Sedation Scale; IQR, interquartile range.

	Group 1 (n= 26)	Group 2 (n=30)	p-value
SOFA score at the onset of the weaning procedure (median, IQR)	2.3 (1,4)	2.1(1-3)	0.4
RASS scale ranging from -2 to +1 (%)	76.9	56.7	0.055
RASS score during weaning (median, IQR)	−0.9 (−2,2)	−1.7(−2.5,1)	0.06
Additional analgesia (Fentanyl) required (%)	23.1	53.3	0.045
Duration of mechanical ventilation prior to enrollment, days, (median, IQR)	7.1 (3,10)	5.1 (2,8)	0.4
Duration of the weaning process (hours) (median, IQR)	67.7 (43,79)	80.1 (53,87)	0.710
Duration of mechanical ventilation (days) (median, IQR)	9.3 (4,12)	7.5 (4,10)	0.3
ICU length of stay (days) (median, IQR)	23 (15,31)	18 (11,27)	0.06
Length of hospital stay (days) (median, IQR)	28(17,37)	22.5 (16,31)	0.06
Norepinephrine, maximum dose (μg/kg/h) (median, IQR)	0.8 (0.1,1.2)	0.68 (0.05,0.85)	0.8
Antipsychotic drugs use ( %)	15.4	53.3	0.01
Duration of sedative drug infusion post-extubation, hours, (median, IQR)	98 (50,138)	12 (3,8)	0.01

In 15 patients (57.7%) treated with dexmedetomidine, the drug was discontinued after the withdrawal of mechanical ventilation. The remaining 11 patients (42.3%) received dexmedetomidine for a median of 98 hours after successful weaning from mechanical ventilation; none of the patients receiving dexmedetomidine had respiratory depression while being on spontaneous ventilation.

**Figure 1:**
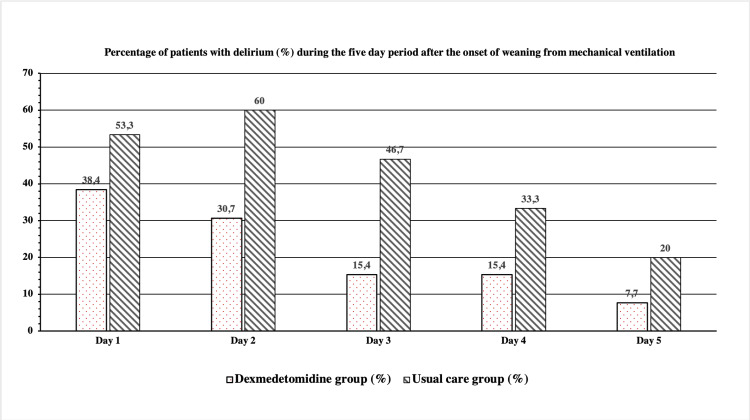
Percentage of patients with delirium during five days after the onset of weaning among the dexmedetomidine and usual care groups. Day 1 is defined as the first day of the weaning process.

Patients who received dexmedetomidine had a lower delirium rate in comparison to patients from the usual care group during the five days after the onset of the weaning process, p=0.02 (Figure 1). Regarding the adverse effects, three patients in the dexmedetomidine group developed bradycardia and one patient developed abdominal distention. These adverse events were reversible and treated by lowering the dosage of the drug.

## Discussion

This retrospective clinical trial evaluated the effect of dexmedetomidine versus propofol and midazolam on clinical outcomes in patients with severe burn injuries receiving mechanical ventilation. Sedation, analgesia, and prevention of delirium are integral parts of the complex management of critically ill burn patients aimed to facilitate mechanical ventilation and reduce patients’ discomfort and anxiety. There is increasing evidence that deep sedation is associated with a high delirium rate, prolonged length of ICU and hospital stay, and increased costs and mortality in critically ill patients [3,19,20]. The most recent guidelines of the Society of Critical Care Medicine regarding pain agitation/sedation, delirium, immobility, and sleep disorders (PADIS recommendations) suggest that the choice of a sedative agent in an ICU setting should be driven by the specific indications and sedation goals for each patient, specific pharmacokinetic and pharmacodynamic characteristics of the drugs, including the side effect profile, and the overall cost associated with the use of the sedatives [2]. The guidelines highlight the need to maintain light sedation in all patients receiving mechanical ventilation and recommend using dexmedetomidine or propofol sedation and avoiding the use of benzodiazepines; however, given the low quality of available evidence, experts deem this recommendation as conditional.

The aim of this trial was to study the optimal way to promote light sedation and facilitate early weaning from mechanical ventilation in Burn ICU patients by evaluating the impact of light sedation with dexmedetomidine versus usual care with either propofol or midazolam on outcome parameters. Our results demonstrated that administration of dexmedetomidine in critically ill burn patients during the weaning from mechanical ventilation was not associated with a significant increase in the number of ventilator-free days or better control of target sedation compared with the usual sedatives. However, the use of dexmedetomidine was associated with lower rates of delirium with minimal and reversible adverse effects.

Dexmedetomidine has been proposed to reduce the duration of mechanical ventilation [3]; however, studies show contradicting results regarding this outcome [7,21,22]. Our results indicated that dexmedetomidine was not associated with decreased duration of mechanical ventilation when compared with usual sedation regimens. Although the use of dexmedetomidine leads to faster weaning from mechanical ventilation (patients on dexmedetomidine were weaned almost 12 hrs earlier), the difference between the dexmedetomidine group and the usual care group was not significant. Our results reflect the conclusions from recent systematic reviews and meta-analyses indicating that the use of dexmedetomidine has no significant effect on the duration of mechanical ventilation in critically ill patients [21]. Jakob et al. analyzed data from two randomized controlled trials and revealed that the use of dexmedetomidine was not inferior to usual care (midazolam and propofol) in maintaining light to moderate sedation; moreover, dexmedetomidine reduced the duration of mechanical ventilation and improved patients’ ability to communicate pain, compared with usual care [22]. However, more instances of adverse events (hypotension and bradycardia) were observed in patients with dexmedetomidine compared to midazolam patients in this study. Reade et al. in a randomized trial investigated the effects of dexmedetomidine in addition to standard care on ventilator-free time in critically ill patients in which agitative delirium was the main factor preventing weaning and reported that the addition of dexmedetomidine to standard care compared with standard care alone was associated with more ventilator-free hours at seven days following randomization and an accelerated resolution of delirium (median, 23.3 hours vs 40.0 hours; p =0.01), thus supporting our findings suggesting reduced rates of delirium in burn patients [7].

Both groups of patients included in our study reached the sedation level goals required for ICU patients. Although patients in the dexmedetomidine group had a higher median RASS score during the weaning procedure (lighter sedation), no statistically significant differences were found between groups of patients taking dexmedetomidine and a placebo (p=0.06). Additionally, our results show a higher percentage of patients with light sedation (mean RASS score scale from -2 to +1) in the dexmedetomidine group (76.9%), in comparison to the usual care group (56.7 %). However, the difference in patients who achieved light sedation was on the cusp of statistical significance (p=0.055). Thus, when considering the level of sedation in ICU burn patients, dexmedetomidine could be more effective in achieving light sedation than other sedative agents; however, there is a need for further evaluation of this association.

Delirium is common during prolonged ICU stays, and sedation itself may be an independent risk factor for the development of delirium [23]. The results of our study indicated that dexmedetomidine was associated with a lower prevalence of delirium in patients receiving the agent compared to standard care in patients during the weaning process. These results are consistent with data from the randomized trials comparing the effect of dexmedetomidine versus benzodiazepines or propofol on delirium rates, which also concluded that dexmedetomidine was associated with less delirium in mechanically ventilated ICU patients [24].

Dexmedetomidine demonstrates analgesic and opioid-sparing effects in critically ill patients in an intensive care setting [25]. In our study, a significantly lower percentage of patients in the dexmedetomidine group received intercurrent opioids; additionally, more patients in the usual care group received antipsychotic medications (haloperidol, risperidone, or quetiapine) than in the dexmedetomidine group. Our data support the findings from the previous study of Reade et al. [7] that dexmedetomidine, when added to standard care, compared with standard care alone, is associated with significantly lower quantities of intercurrent sedatives and opioids. Another study focusing on the effects of dexmedetomidine in patients undergoing open cardiac surgery confirmed our findings and demonstrated the decreased need for supplemental analgesia with dexmedetomidine in comparison to propofol sedation [26].

Data suggest that the use of dexmedetomidine may be associated with a higher rate of hypotension and bradycardia [27]. In our patients, the use of dexmedetomidine was well tolerated with minimal reversible adverse effects including bradycardia and abdominal distention. It is worth noting that a high percentage of patients continued to receive dexmedetomidine after liberation from mechanical ventilation and none of them had respiratory depression after a successful weaning procedure. We did not observe hemodynamic compromises (hypertension or hypotension episodes) related to the use of dexmedetomidine and there were no differences in vasopressor requirements between the two groups of patients. Our data confirmed the recent evidence from a subgroup analysis of the sedation practice in intensive care (SPICE III) trial which stated that in critically ill patients with septic shock, compared to usual care, patients receiving early sedation with dexmedetomidine as the primary sedative agent had similar vasopressor requirements in the first 48 h compared to usual care. Moreover, multivariable adjusted analysis revealed that dexmedetomidine appeared to be associated with lower vasopressor requirements to maintain the target MAP [28].

A retrospective chart review of 65 pediatric burn ICU patients who received dexmedetomidine infusion because of failure to achieve adequate sedation with standard sedation and analgesia regimen demonstrated that prolonged use of dexmedetomidine in pediatric burn patients (the average duration of dexmedetomidine infusion of 11 days, range 2-50) was not associated with evidence of tachyphylaxis, rebound hypertension or adverse withdrawal effects [29]. Moreover, the use of dexmedetomidine was safe and did not induce respiratory depression in extubated patients. Fagin et al. compared dexmedetomidine and midazolam for prolonged sedation in pediatric patients with severe burn injury (mean duration (SD) of infusion was 22.5 (24.9) days for dexmedetomidine and 20.1 (24.8) days for midazolam) and concluded that patients with dexmedetomidine had higher Richmond Agitation Sedation scale scores in comparison with the midazolam group (a mean of RASS of -0.91 ± 0.8 vs. -1.33 ± 0.7, respectively) [30]. Additionally, patients receiving dexmedetomidine developed fewer sedation-related hypotension episodes and no significant difference in bradycardia episodes was observed compared with patients sedated with midazolam, confirming the results of our study that indicate low rates of adverse effects related to dexmedetomidine sedation.

Study limitations

Our results need to be interpreted carefully. The retrospective character of the study may involve the risk of bias in the estimation of the overall treatment effect and outcomes within the two study groups, more specifically, dexmedetomidine patients were younger and had a higher percentage of 3rd-degree burn injuries. Additionally, due to the retrospective nature of the study, the presence of potential confounding factors that are unavailable in electronic and written records could not be controlled. Additionally, due to the small number of patients, a decision was made not to perform a separate analysis of the effects of propofol and midazolam in comparison with dexmedetomidine.

## Conclusions

The results of our study reflect the current literature evidence that dexmedetomidine may provide effective light sedation and is associated with few sedation-related adverse effects in burn patients. However, dexmedetomidine use during the weaning process in adult burn patients did not seem to lower the duration of mechanical ventilation. Dexmedetomidine sedation was associated with a lower delirium rate in comparison with usual care agents and was associated with a trend towards the earlier withdrawal of mechanical ventilation. No differences were observed in the use of vasopressors between the dexmedetomidine and standard care groups. The use of dexmedetomidine compared with usual care resulted in lower quantities of intercurrent analgesia and antipsychotic drugs and a low rate of complications. The findings of this retrospective study justify the need for a large confirmatory randomized trial to evaluate whether goal-directed sedation with dexmedetomidine influences outcome characteristics in Burn ICU patients.
